# TLR-4/Wnt modulation as new therapeutic strategy in the treatment of glioblastomas

**DOI:** 10.18632/oncotarget.26500

**Published:** 2018-12-25

**Authors:** Giovanna Casili, Maria Caffo, Michela Campolo, Valeria Barresi, Gerardo Caruso, Salvatore M. Cardali, Marika Lanza, Raffaella Mallamace, Alessia Filippone, Alfredo Conti, Antonino Germanò, Salvatore Cuzzocrea, Emanuela Esposito

**Affiliations:** ^1^ Department of Chemical, Biological, Pharmaceutical and Environmental Sciences, University of Messina, Messina, Italy; ^2^ Department of Biomedical and Dental Sciences and Morphofunctional Imaging, Unit of Neurosurgery, University of Messina, Messina, Italy; ^3^ Department of Human Pathology, University of Messina, Messina, Italy; ^4^ Unit of Anesthesia, University of Messina, Messina, Italy

**Keywords:** glioblastomas, TLR-4, Wnt, Dkk-3, claudin-5

## Abstract

**Purpose:**

Glioblastomas are highly aggressive brain tumors. Various pathways are involved in gliomagenesis, among which the Wingless (Wnt) signaling. Dickkopf protein-related protein 3 (Dkk-3) interacts with proteins of Wnt pathwayas inhibitor. The Wnt signaling contributes to activity of the claudins, that are critical components of tight junctions, whose expression was altered selectively in cerebral microvessels of glioblastoma. The aim of this study was to determine the role of Wnt pathways in the regulation of tumor growth, apoptosis process by targeting Dkk-3, tight junctions alteration involving claudin-5, suggesting possible therapeutic interactions involving Wnt/Toll-like receptors (TLRs) pathways.

**Results:**

We showed a significant decreasing of Dkk-3 and claudin-5 in human glioblastoma cell lines, as well as in U-87 MG xenograft tumors and in glioblastoma human patient’s tissues, with an involvement of the apoptosis process. Also, an interesting TLR-4/Wnt modulation highlighted that the absence of TLR-4 determined resistance to the tumor onset.

**Conclusions:**

We concluded that combined modulation of Wnt/Dkk-3/claudin-5 and TLR-4 pathways, simultaneously targeting apoptosis and survival signaling defects, might shift the balance from tumor growth stasis to cytotoxic therapeutic responses, flowing in greater therapeutic benefits.

**Methods:**

In the present study we investigated the expression of Dkk-3, claudin-5, apoptosis markers and TLR-4 receptor protein levels in *in vitro* studies on U-138MG, A-172, LN-18 and LN-229 human glioblastoma cell lines, and *in vivo* study using TLR-4 KO mice and in glioblastoma human patient’s tissues.

## INTRODUCTION

Glioblastoma (GBM) is the most common primary brain tumor in adults [1], with a dismal prognosis and an incidence rate of approximately 3.19/100,000 per year [2]. Despite significant advances in our basic understanding of tumor pathogenesis, the median overall survival of patients has increased only 3.3 months (from 11.3 months to 14.6 months) over the past 25 years [3]. This poor prognosis is largely due to the recurrence of tumors after initial treatment with maximal safe surgical resection, radiotherapy and chemotherapy. Furthermore, the 5-year survival rate for patients with GBM remains low and innovative therapies are needed [4]. Various pathways are involved in gliomagenesis, among which the Wingless (Wnt) signaling. In malignant glioma, the gene and protein expressions of Wnt were higher than in normal human brain tissues and related to the malignancy grade [5]. However, the role and manner of the interaction of Wnt proteins with the receptors remain unclear. Dickkopf (Dkk) family members exhibit distinct overlapping expression patterns in mouse and human tissues, differently involving Wnt signaling. Dickkopf protein-related protein 3 (Dkk-3) is one of the most promising tumor suppressor molecules able to modulate the Wnt pathway [6]. To date, the most evident and consistent anti-tumor effect of Dkk-3 is its inhibitory capacity on cancer cell growth [7].

The Wnt signaling contributes to the activity of claudins (most prominently claudin-1, claudin-3, and claudin-5), one of the families of proteins which constitute tight junctions (TJ) [8]. Claudin 5 is a key component of the TJ strand, particularly in brain endothelial cells. Its major role is to decrease the permeability to ions. In various pathological processes, including inflammation, edema, toxic damage, trauma and tumor, claudin 5 regulates the change in endothelial or epithelial permeability [9]. Recently, Hara *et al.*, [10] demonstrated that blocking of the interaction between Wnt proteins and their co-receptors contributes to the anti-tumor effects of adenovirus-mediated Dkk-3 in GBM.

The approach to GBM treatment is complicated by the fact thatgliomas escape immune surveillance by creating an immune suppressed microenvironment. One of the more promising therapeutic strategies for the treatment of gliomas is the immunotherapy. Molecules fundamental to eliciting an immune response in various immune cell types are Toll-like receptors (TLRs) [11]. TLRs are an evolutionarily conserved family of pattern-recognition receptors with 10 functional members (TLR 1–10) in human that are expressed on a variety of immune cell types. Toll-like receptor agonists are pathogen-associated molecular patterns (PAMPs) that bind to TLRs with specificity and in a typical scenario, initiate an immune response. Particularly, Toll-Like Receptor 4 (TLR-4) RNA and/or protein expression has been detected in different glioma cell lines [12], in primary biopsies from glioblastoma patients [13] and adjacent to non-neoplastic tissue [14].

The purpose of this study was to evaluate the Wnt-signaling-mediated role of Dkk-3 and claudin-5 in *in vitro* studies on GBM cell lines, in *in vivo* study on TLR-4 KO mice and in a clinical study on 30 WHO grade IV GBM patients. The correlation between Dkk-3 and claudin-5 expression and its decreasing through caspase-dependent apoptosis could represent new interesting therapeutic strategies for GBM treatment.

## RESULTS

### *In vitro* studies

#### Role of Dkk-3 and claudin-5 in human GBM cells

**Figure 1:**
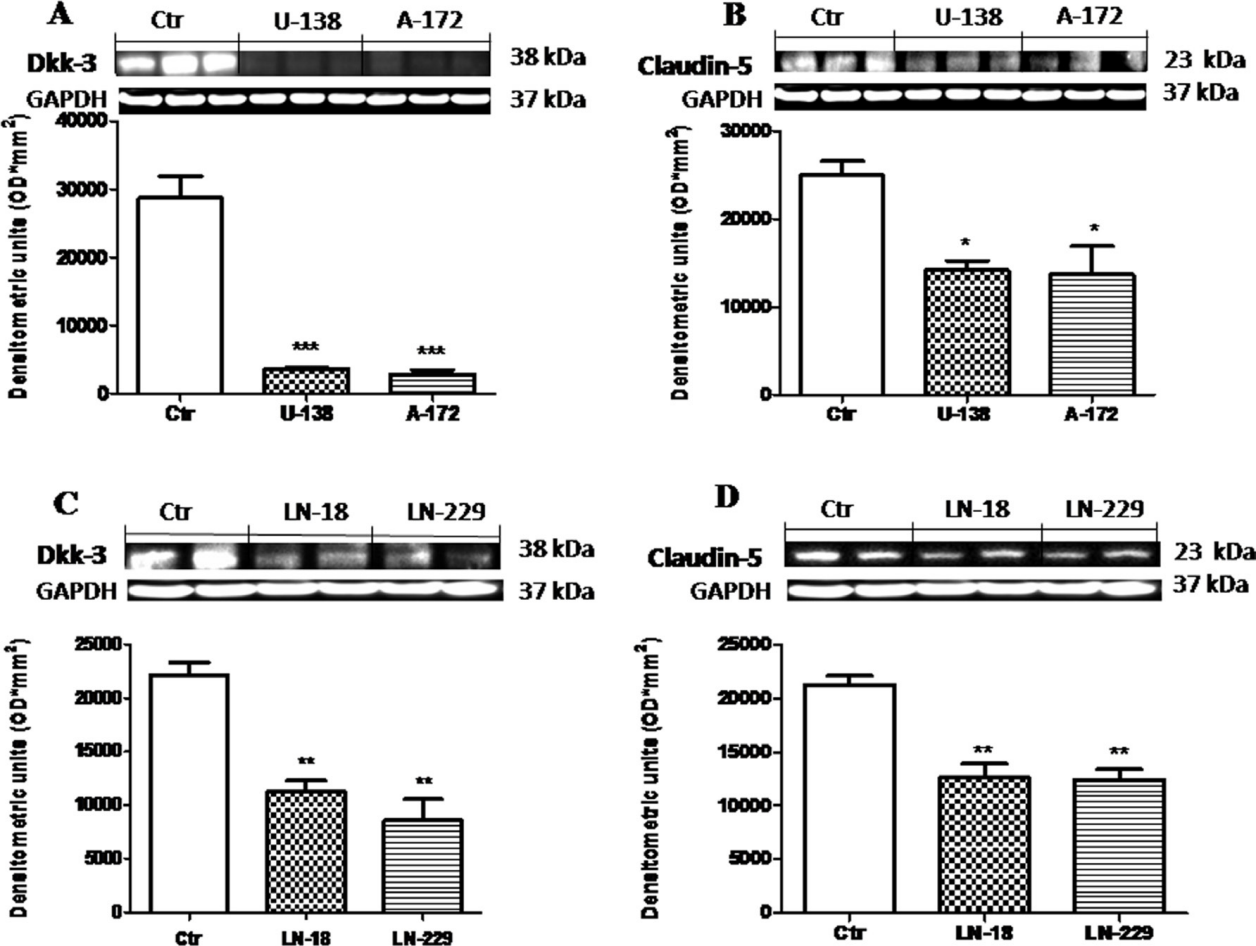
Role of Dkk-3 and claudin-5 in human GBM cells The expression of Dkk-3 was drastically higher from NHA cells compared to human GBM cells (respectively **A** and **C**). Similarly, expression of claudin-5 was moderately lower in U-138MG, A-172, LN-18 and LN-229 cell lines, while NHA cells significantly expressed claudin-5 (respectively **B** and **D**). Each data are expressed as mean ± SEM. One-Way ANOVA test followed by Bonferronipost test. ^*^p < 0.05, ^**^p < 0.01 and ^***^p < 0.001 vs Ctr. Dkk-3 and claudin-5 in U138MG and A172 were detected in the same membrane (**A** and **B**) as well as for Dkk-3 and claudin-5 in **C** and **D**. Therefore, the ubiquitary GAPDH band is the same for Dkk-3 and claudin-5 (**A**–**B** and **C**–**D** respectively).

To better investigate the way in which Wnt signaling modulate Dkk-3 protein and claudin-5 expression we examined the expression of Dkk-3 and claudin-5 in human GBM cell lines (U-138MG, A-172, LN-18 and LN-229), using NHA cells as control, by Western Blot analysis. We found that the expression of Dkk-3 was drastically higher in NHA cells used as control compared to human GBM cells (Figure 1A and 1C). Similarly, claudin-5 expression was significantly lower in U-138MG, A-172, LN-18 and LN-229 cell lines, while NHA cells significantly expressed claudin-5 (Figure 1B and 1D). Each data are expressed as mean ± SEM. One-Way ANOVA test followed by Bonferroni post test. ^*^p < 0.05, ^**^p < 0.01 and ^***^p < 0.001 vs Ctr.

### Dkk-3 modulation of apoptosis in human GBM cells

**Figure 2:**
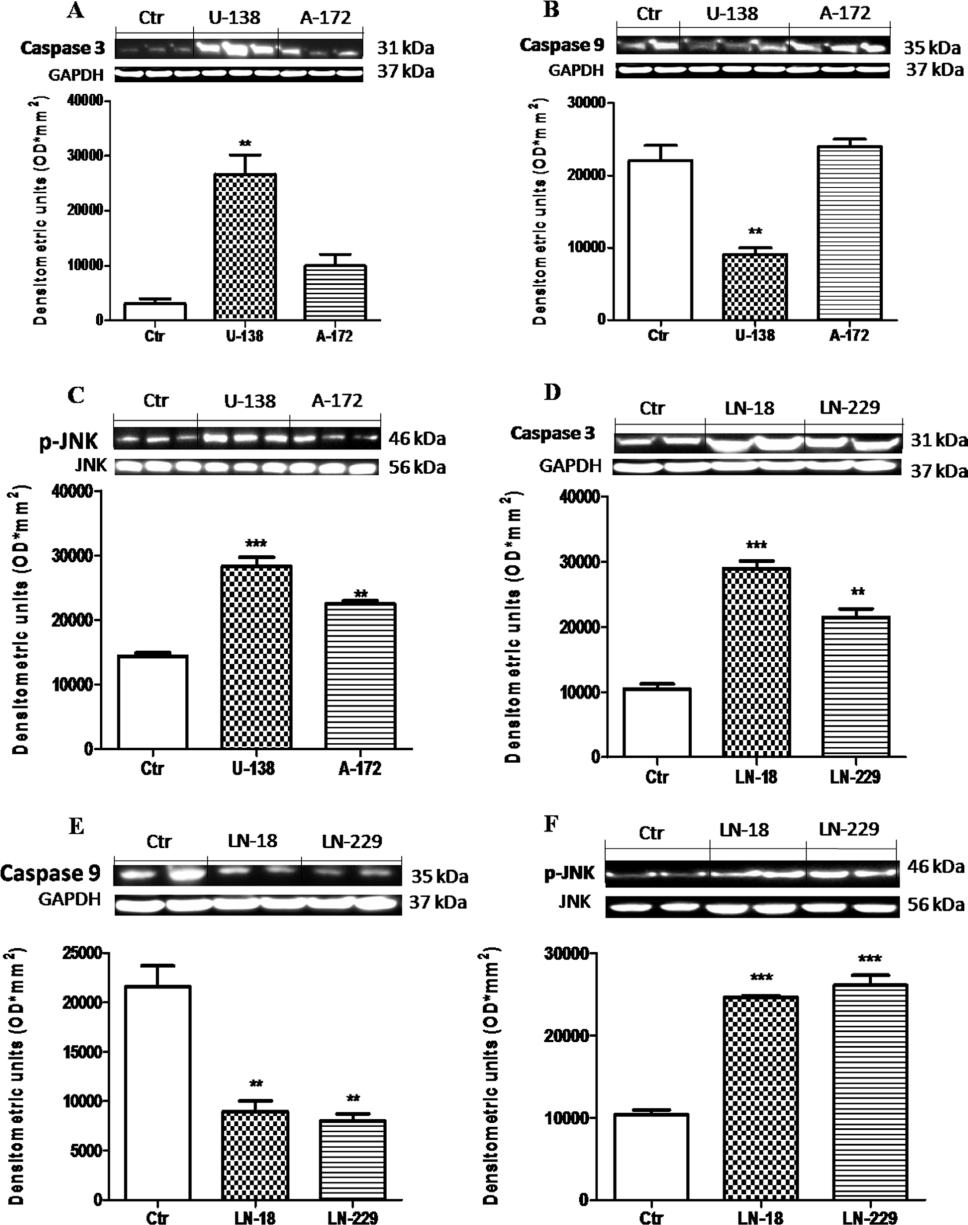
Apoptosic process in human glioblastoma cells A significant GBM cells apoptosis-resistance was observed in U-138MG, LN-18 and LN-229 cells, notably reduced in NHAcells (**B** and **E**). Conversely, Caspase-3 expression was significantly reduced in NHA cells, with a rising trend in A-172 and L-229 andsignificantly higher in U-138MG and LN-18 cells (**A** and **D**). Also, p-JNK expression was significantly higher in U-138MG, A-172, LN-18 and LN-229 cell lines, while lower p-JNK expression was highlighted in NHA astrocytes (**C** and **F**). Each data are expressed as mean ± SEM. One-Way ANOVA test followed by Bonferronipost test. ^*^p < 0.05, ^**^p < 0.01 and ^***^p < 0.001 vs Ctr. Caspase 3 (**A**) was detected in a stripped membrane. Therefore, the ubiquitary GAPDH band is the same for Dkk-3 and claudin-5 in Figure 1 (**A** and **B** respectively).

The potential antitumoral effects of a new therapeutic strategy are often established evaluating tumor cells apoptosis. Caspases are key mediators in the regulation and execution of apoptosis. Caspase-9 is activated during the intrinsic pathway while Caspase-3 is an effector caspase that initiates degradation of the cell in the final stages of apoptosis. We hypothesized that the downregulation of Dkk-3 could promote apoptosis, providing a potential strategy for GBM treatment. So, to testify how Dkk-3 could increase apoptosis processes, we analyzed the expression of Caspase-3 and Caspase-9 protein, apoptotic and anti-apoptotic proteins respectively, in GBM cell lines by Western Blot analysis. We observed a basal expression of Caspase-3 in NHA cells, while a considerable increase was observed respectively in U-138MG andA-172 cell lines (Figure 2A) and in LN-18 and LN-229 (Figure 2D). Conversely a decrement in Caspase-9 levels was significantly shown in U-138MG cells, while A-172 cells displayed a Caspase-9 expression similar to control cells (Figure 2B). LN-18 and L-229 cell lines showed a decrement in Caspase-9 expression, respect to NHA control cell lines (Figure 2E). Please note that U-138MG and A-172 cell lines responded to apoptotic processes in different way. Since it is known that Dkk-3 acts as tumor suppressor but did not inhibit the Wnt/β-catenin signaling pathway, rather inducing apoptosis via the noncanonical JNK pathway [15], we also evaluated the p-JNK expression in GBM cell lines. We observed that p-JNK expression was significantly higher in U-138MG and A-172 cell lines (Figure 2C) and in LN-18 and LN-229 cells (Figure 2F). Each data are expressed as mean ± SEM. One-Way ANOVA test followed by Bonferroni post test. ^*^p < 0.05, ^**^p < 0.01 and ^***^p < 0.001 vs Ctr.

### Role of TLR-4 in the modulation of Wnt/Dkk-3 axis

**Figure 3:**
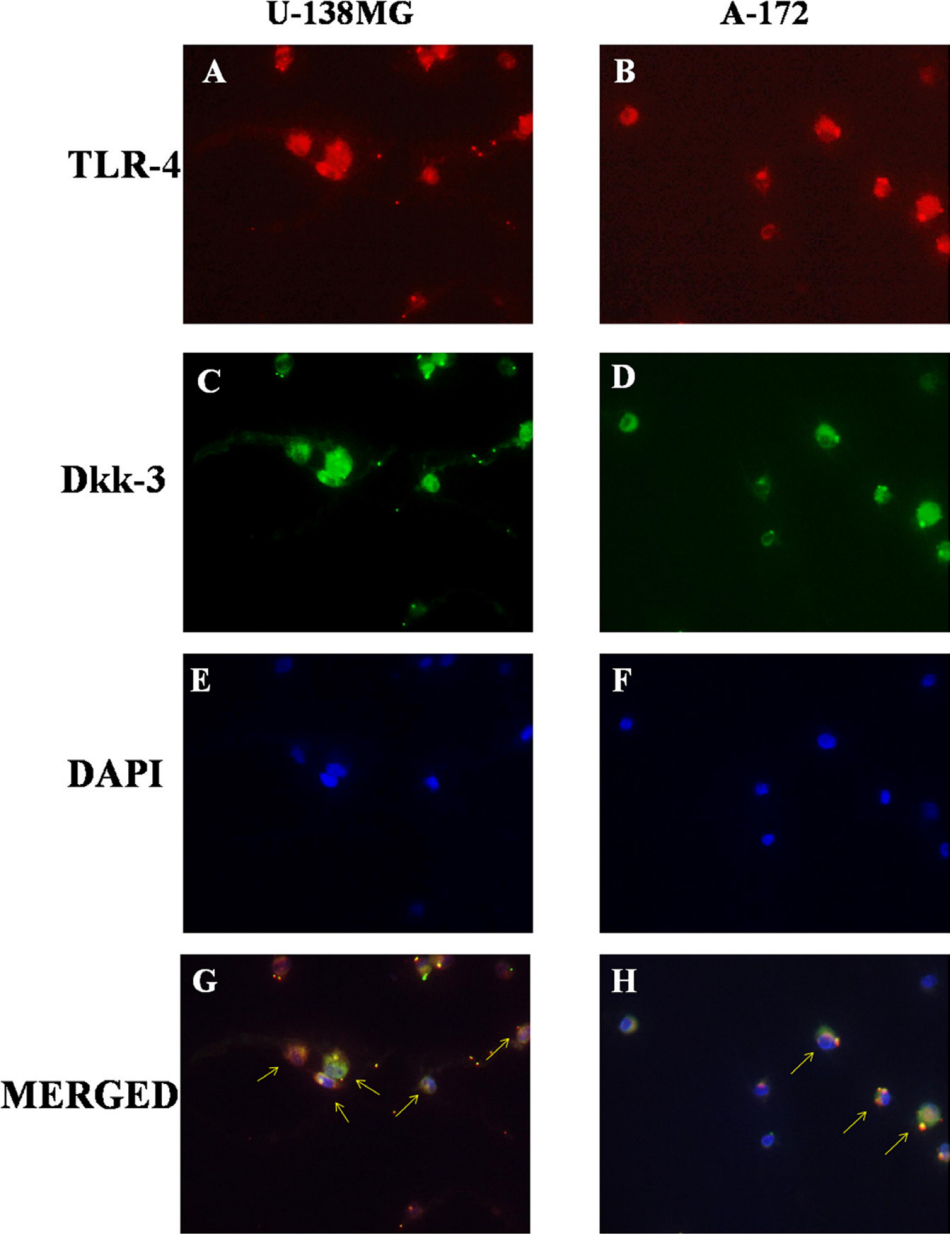
Role of TLR-4 in the modulation of Wnt/Dkk-3 axis A diffuse expression of both TLR-4 and Dkk-3 in U-138MG (**A** and **C**) and in A-172 (**B** and **D**) cell lines was observed by immunofluorescence analysis. Cell nuclei were stained in blue with DAPI (**E** and **F**). Yellow spots are indicative of a significant TLR-4/Dkk-3 co-localization (**G** and **H**).

The possibility that TLR-4 may activate Wnt signaling was recently evaluated, suggesting that TLRs mediate many of these functions in immune cell types and are currently possible primary candidate molecules to be used as important adjuvants in a variety of cancers [11]. So, to better understand the role of TLR-4 in Wnt signaling on GBM, binding with Dkk-3, we analyzed TLR-4/Dkk-3 co-localization by immunofluorescence analysis in GBM human cell lines. We observed a significant expression of both TLR-4 and Dkk-3 in U-138MG (Figure 3A and 3C) and in A-172 (Figure 3B and 3D) cell lines. Cell nuclei were stained in blue with DAPI (Figure 3E and 3F). Yellow spots are indicative of a significant TLR-4/Dkk-3 co-localization (Figure 3G and 3H).

### Evaluation of DNA damage in human GBM cell line

**Figure 4:**
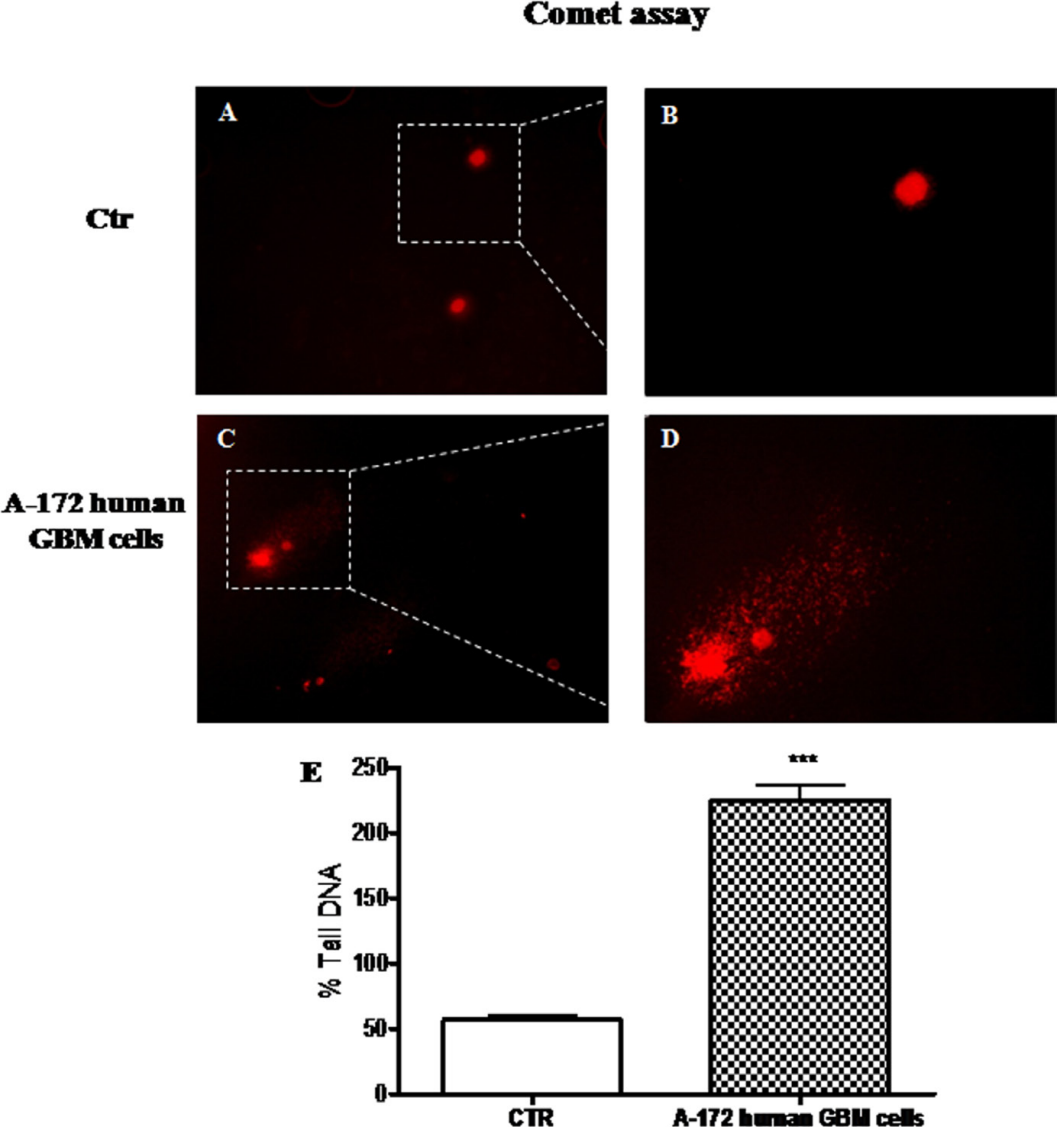
The role of DNA damage in GBM human cell lines Comet assay on GBM human cell lines. Significant increase in the % tail DNA pattern in A-172 human GBM cells (**C** and **D**, see % Tail DNA graph **E**), respect to NHA cells used as control, with an intact heads (**A** and **B**, see % Tail DNA graph **E**). Each data are expressed as mean ± SEM. *T*-test followed by unpaired test. ^***^p < 0.001 vs Ctr.

DNA damage and defective DNA repair, measured as the change in % tail DNA, were evaluated performing comet assay on A-172 human GBM cells and on NHA cells, used as control. We observed a significant increase in the % tail DNA pattern in A-172 human GBM cells (Figure 4C and 4D, see % Tail DNA graph Figure 4E), respect to NHA cells used as control, that showed an intact heads (Figure 4A and 4B, see % Tail DNA graph Figure 4E).

### *In vivo* studies

#### Role of TLR-4 in tumor growth

**Figure 5:**
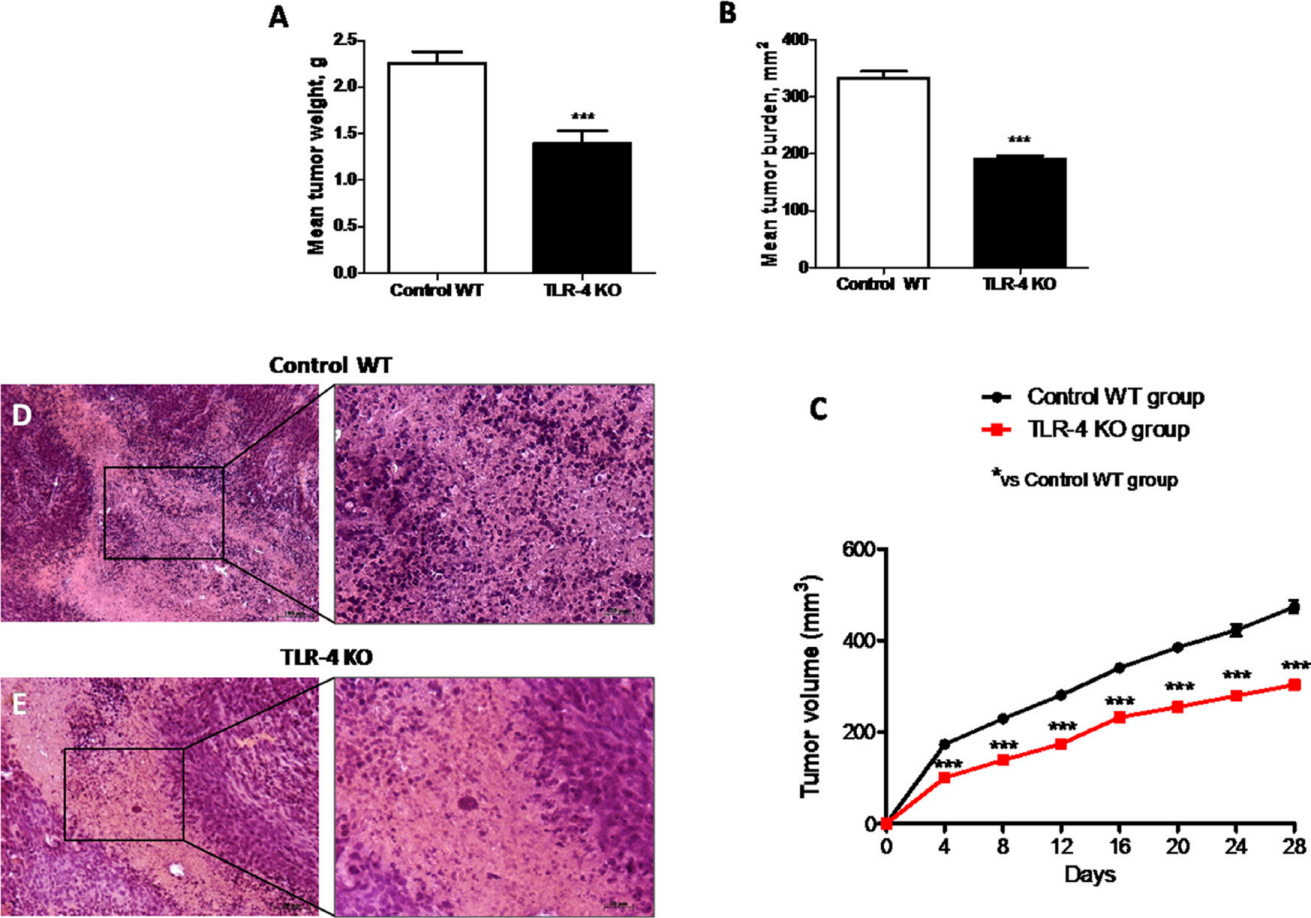
Effects of TLR-4 on tumor growth Effect of TLR-4 absence on mean tumor weight (**A**) and tumor burden (**B**) in nude mice grafted with U-87 MG cells. During the 28-days observation period, tumor growth was significantly reduced in TLR-4 KO mice respect to Control WT mice (**C**). The histological tumor evaluation highlighted necrosis and associated neutrophilic inflammation more present in tumors from Control WT mice respect to TLR-4 KO tumor sections (**D** and **E**). Each data are expressed as mean ± SEM. *T*-test followed by unpaired test and Two-way ANOVA followed by Bonferroni post test. ^***^p < 0.001 vs Ctr.

Activation of the innate immune system, particularly through TLR-4, may stimulate tumor growth. Also, the expression of TLR-4 on tumor cells was reported to play a role in immune surveillance and facilitate tumor growth and chemoresistance [16]. In this study, we observed that the absence of TLR-4 strongly inhibited the growth of U-87 MG xenografts in mice. Mean tumor weight was significantly reduced (*p* = 0.9701) as shown in Figure 5A, and tumor burden was inhibited by 42% (*p* = 0.128), as shown in Figure 5B, suggesting TLR-4 KO mice showed a greater resistance to the tumor onset and development. Also, during the 28-days observation period, tumor growth was significantly inhibited in TLR-4 KO mice compared to Control WT mice, as shown in Figure 5C (*F* value = 20.77). Moreover, tumors sections from control mice, stained with hematoxylin – eosin (H/E) showed subcutaneous masses, composed of solid sheets and nests of irregularly round epithelioid cells with prominent round to ovoid nuclei with ill-defined cell borders, as well as an increase of tumor necrosis and neutrophilic infiltration; indeed TLR-4 KO mice showed a significantly reduction of tumor sections (10–60% in TLR-4 KO compared to 50–80% from Control WT mice) as well as in neutrophil infiltration as shown in Figure 5E. Each data are expressed as mean ± SEM. *T*-test followed by unpaired test and Two-way ANOVA followed by Bonferroni post test. ^***^p < 0.001 vs Ctr.

### Effects of TLR-4 on Dkk-3 and claudin-5 expressions in U-87 xenograft tumor

**Figure 6:**
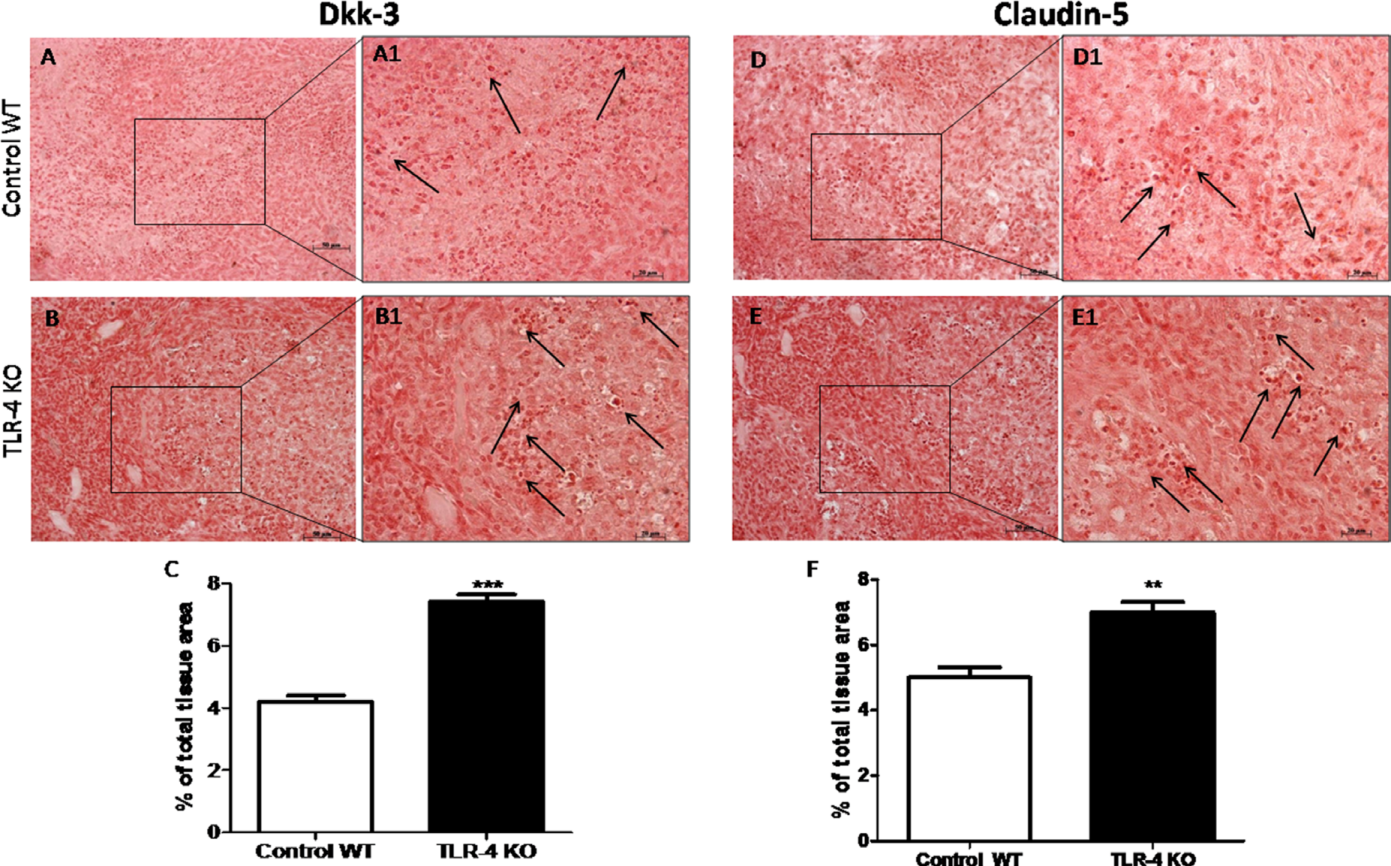
Effects of TLR-4 on Dkk-3 and claudin-5 expressions in U-87 xenograft tumors A remarkable higher positive staining for Dkk-3 and claudin-5 on TLR-4 KO brain sections was shown (**B**–**B1** and **E**–**E1**, see graphs respectively **C** and **F**) compared to control WT section (**A**–**A1** and **D**–**D1**, see graphs respectively **C** and **F**). Black spots are indicative of a significant Dkk-3 (**A1** and **B1**) and claudin-5 (**D1** and **E1**) expressions. Each data are expressed as mean ± SEM. *T*-test followed by unpaired test. ^**^p < 0.01 and ^***^p < 0.001 vs Control WT.

The role of Dkk-3 as a tumor suppressor has been recently suggested [11]. To better understand if the gene deficiency of TLR-4 could increase the expression of Dkk-3, we made an immunoistochemical analysis of Dkk-3 and claudin-5 on tumor sections. An evident positive staining for Dkk-3 and claudin-5 was shown on TLR-4 KO tumor brain sections (Figure 6B–B1 and 6E–E1, see graphs respectively Figure 6C and 6F) compared to control WT section (Figure 6A–A1 and 6D–D1, see graphs respectively Figure 6C and 6F). Black arrows are indicative of a significant Dkk-3 (Figure A6 and B6) and claudin-5 (Figure D6 and E6) expressions. *T*-test followed by unpaired test. ^**^p < 0.01 and ^***^p < 0.001 vs Control WT.

### Effects of TLR-4 on apoptosis in U-87 xenograft tumors

**Figure 7:**
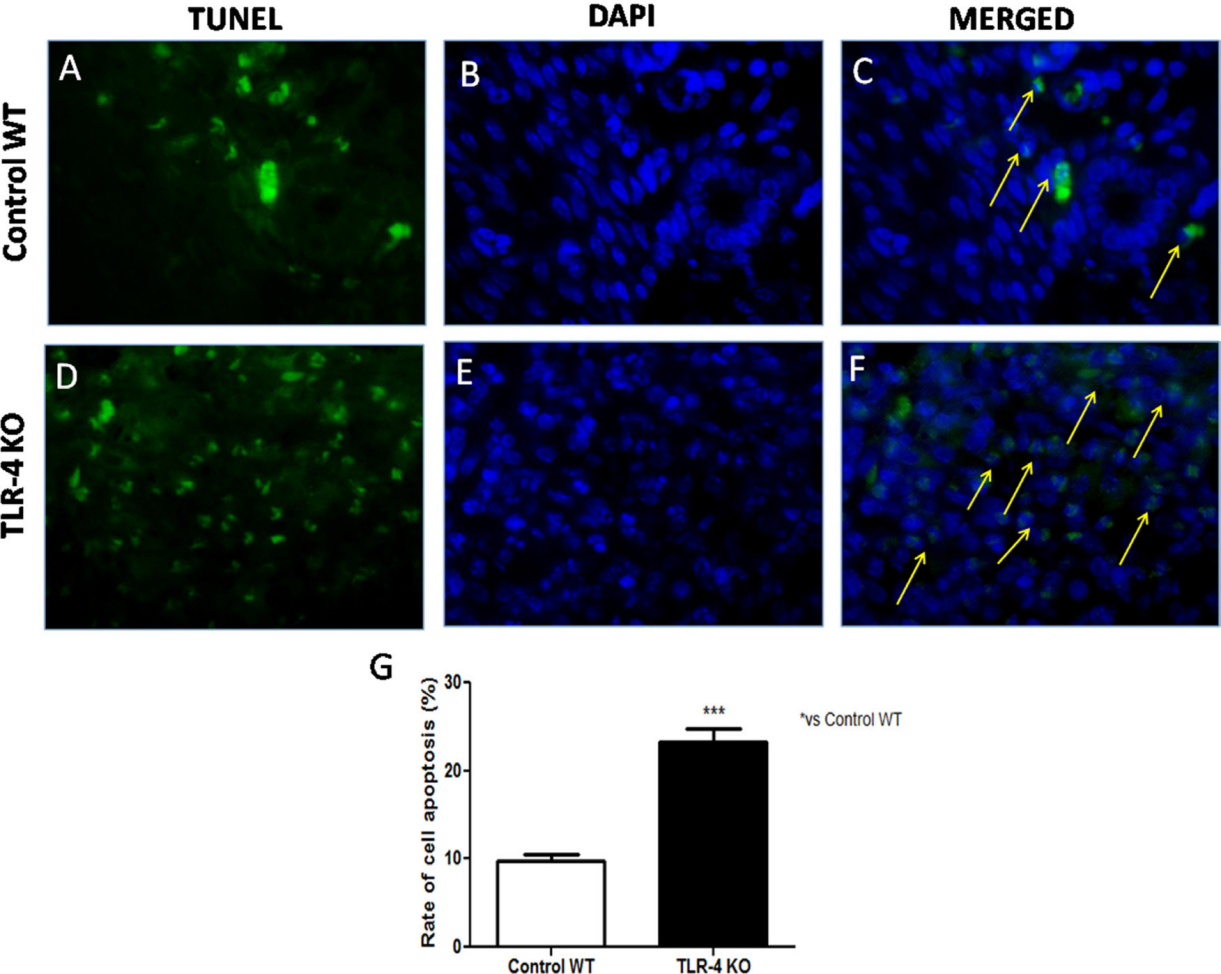
Effects of TLR-4 on apoptosis in U-87 xenograft tumors Representative images of the TUNEL assay (green fluorescence of apoptotic cells and blue fluorescence of cell nuclei) on control WT tumor sections (**A**, **B** and **C**) and on TLR-4 KO tumor sections (**D**, **E** and **F**). The rate of cell apoptosis of each group was evaluated (**G**). Each data are expressed as mean ± SEM. *T*-test followed by unpaired test ^***^p < 0.001 vs Ctr WT.

He *et al.*, demonstrated that TLR-4 expressed on human cancer cells is functionally active and plays important roles in promoting immune escape of human cancer cells by inducing immunosuppressive cytokines and apoptosis resistance [11]. Thus, to understand if the lack of TLR-4 could increase apoptosis in U-87 xenograft tumors, TUNEL staining was performed on tumor sections from tumor sample taken after 28 days. Few TUNEL-positive apoptotic cells were found in Control WT tumor sections (Figure 7A, 7B and 7C, see graph Figure 7G), while TLR-4 absence determined a significant increase in TUNEL positive cells on tumor sections (Figure 7D, 7E and 7F, see graph Figure 7G). The rate of cell apoptosis of each group was evaluated (Figure 7G). Each data are expressed as mean ± SEM. *T*-test followed by unpaired test ^***^p < 0.001 vs Ctr WT.

### Clinical studies

#### Clinical data

Our series includes 30 patients, 9 women and 21 men, aged between 46 and 65 years with an average age of 52.5%. The neoplasm was typically located in the supratentorial region: frontal lobes (four cases), temporal lobes (three cases), parietal lobes (three cases), occipital lobes (two cases). In ten cases, GBM showed a multi-lobar localization: fronto-tempo-parietal lobes (five cases), fronto-temporal lobes (four cases), parieto-occipital lobes (two case). Four cases were located in the temporo-insular region, and the remaining three cases in the thalamic region.

Patient clinical presentation can vary greatly depending on the size and location of the tumor and the anatomic structures of the involved brain. It is also important to take into the account of surrounding edema from the tumor itself as it can cause more neurological deficit than the tumor itself. Preoperative symptoms included headache, nausea, seizures, hemiparesis, visual disturbance, stroke-like symptoms, memory problems, or personality changes. Six patients showed symptoms of increased intracranial pressure. The neurological evaluation showed hemiparesis, positive Babinski sign, visual disturbance with papilledema.

**Figure 8:**
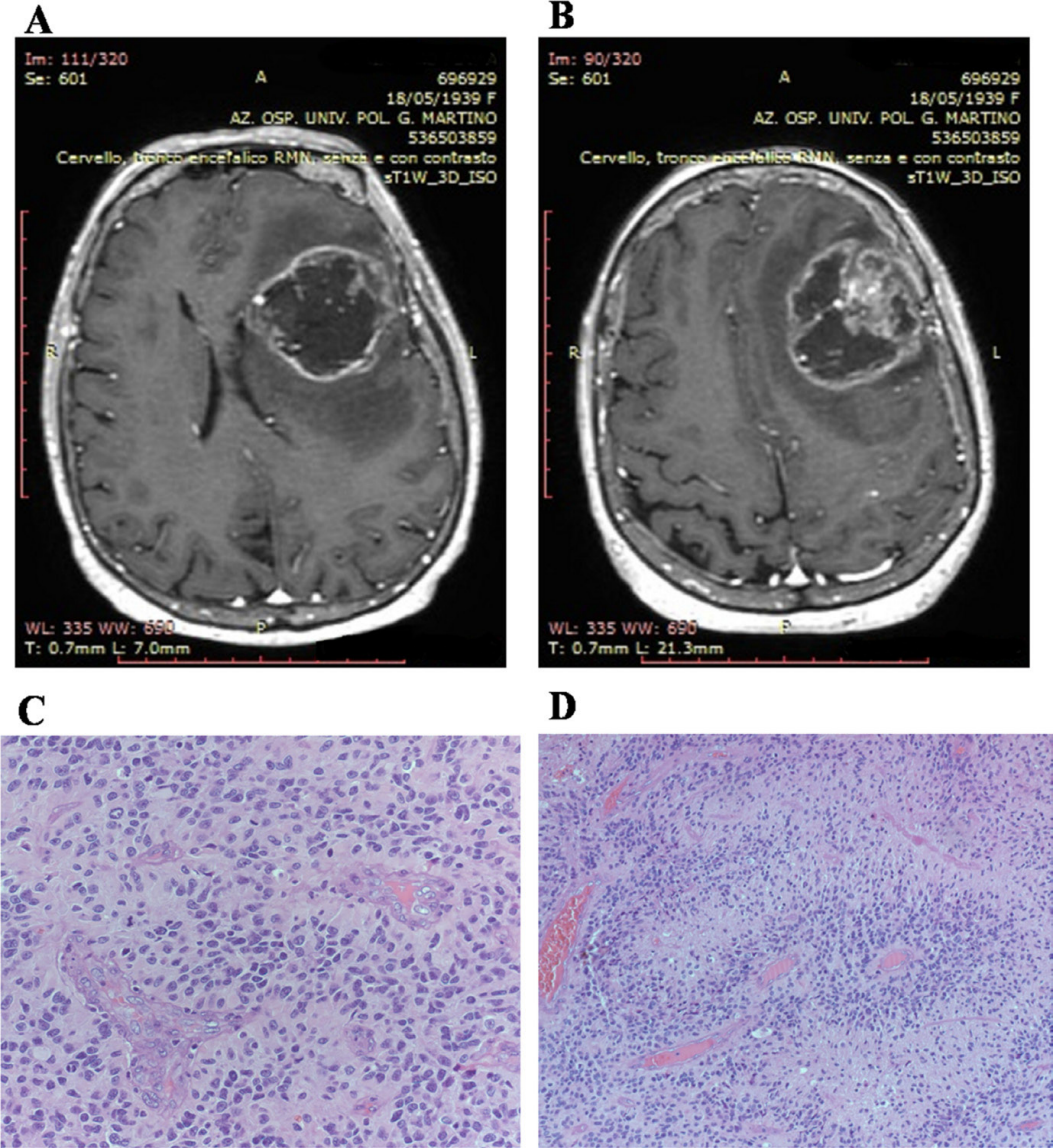
Patients magnetic resonance study and histologic features of biopsies Magnetic resonance study shows, in axial view, on T1-weighted images, an irregular left fronto-temporal lesion with a large, hypointense, necrotic area. The lesion appears surrounded by a peripheral, irregular, enhanced wall after gadolinium administration. The lesion appears surrounded by edema that contributes to the compressive effect that the lesion exerts on the surrounding cerebral areas (**A**); Magnetic resonance study shows, in axial view, on T1-weighted images, an irregular left fronto-temporal lesion. Shift of the midline structures is highlighted. In the context of the hypointense area, an important nodular component is visible (**B**). Palisanding necrosis in glioblastoma (Haematoxylin and eosin stain; original magnification, ×100) (**C**). Glomeruloid microvascular proliferation and brisk mitotic activity in glioblastoma (Haematoxylin and eosin stain; original magnification, ×200) (**D**).

All patients undergone MR study. At MR with gadolinium contrast, GBM typically appear as a contrast enhancing mass, with a thickened ring of enhancement and a hypointense core, which usually corresponds to central areas of necrosis. Necrosis is a hallmark feature of GBM according to the World Health Organization classification system (WHO). The margins of the tumor may be irregular or poorly defined, with spread of the tumor along white matter tracts. Traditional MRI is also capable of demonstrating the degree of edema surrounding a tumor, and susceptibility imaging can show whether or not a tumor contains micro-hemorrhages (Figure 8A and 8B).

A needle biopsy was performed in those cases (five) characterized by unresectable tumors due to their deep localization or owing to the patients were affected by multiple co-morbidities. We adopted a stereotactic system with a frameless guided needle system to obtain tumoral tissue. In the remaining cases, a standard craniotomy approach, with the patient under general anesthesia, was performed. We used in all cases the intra-operative navigation systems, thus being able to better determine the tumor location and plan the incision and craniotomy accordingly.

Surgery allows histologic confirmation of the diagnosis as well as cytoreduction. Surgery may also serve a therapeutic role by reducing the intracranial pressure, and depending on the location of the tumor, occasionally leads to recovery of some neurological function. When maximal resection is not feasible (90% of resection), supramarginal resection of the tumor is an option, which is defined as doing resection beyond tumor mass enhancement displayed by the imaging techniques. In our patients, we have achieved the maximum resection in seventeen cases; in the remaining eight we have obtained a supramarginal resection. Fluorescence-guided surgery and indocyanine green were selectively adopted during surgical procedure. Intercellular area of necrosis, endothelial hyperplasia, and mitotic figures were evidenced in all specimens. The majority of cells shown small dark nuclei and multiple fibrillary processes (Figure 8C and 8D). Secondary structures such us pseudopalisanding were, also observed in a large number of cases. Twenty-eight patients are still alive, two died. One patient died for pneumonia during chemotherapy. The remaining patient died for disease progression. All patients underwent Stupp protocol with radiotherapy and chemotherapy with Temozolomide. Every two months we submit all patients to MR to evaluate response to treatments. The live patients are all still in follow-up.

### Role of Dkk-3 and claudin-5 in GBM patients

**Figure 9:**
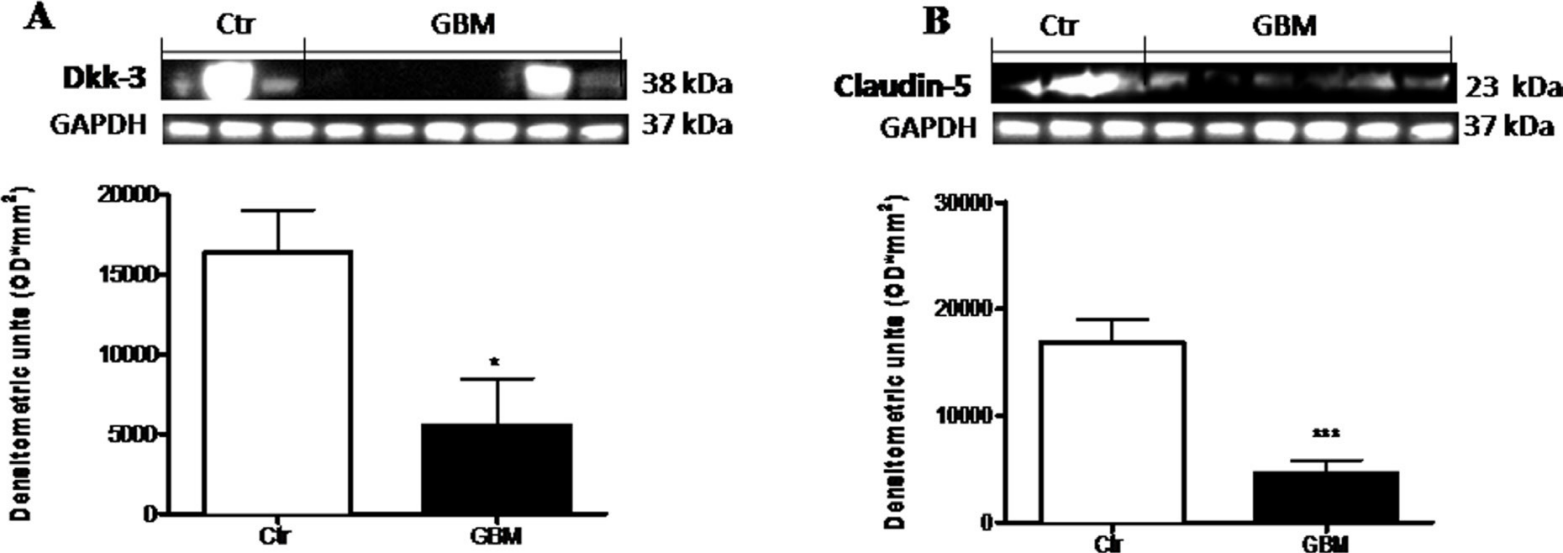
Role of Dkk-3 and claudin-5 in human GBM cells A considerable Dkk-3 expression was observed in controls compared to GBM patients (**A**). Similarly, claudin-5 expression was moderately lower in patients with tumor, while healthy people significantly expressed claudin-5 (**B**). Each data are expressed as mean ± SEM of patients. *T* test, Unpaired *T* test. ^*^p < 0.05 and ^***^p < 0.001 vs Ctr. Both antibodies were detected in the same membrane by using stripping method. Therefore, the ubiquitary GAPDH band is the same for Dkk-3 and claudin-5 (**A** and **B** respectively).

We examined the expression of Dkk-3 protein and claudin-5, in the light of their critical role in the GBM progression. The expression of Dkk-3 and claudin-5 were analyzed by Western Blot analysis in GBM patients respect to healthy people. A considerable Dkk-3 expression was observed in controls compared to GBM patients (Figure 9A). Similarly, claudin-5 expression was moderately lower in patients with tumor, while healthy people significantly expressed high claudin-5 levels (Figure 9B). Healthy human brain tissues were used as control. Each data are expressed as mean ± SEM of patients. *T* test, Unpaired *T* test. ^*^p < 0.05 and ^***^p < 0.001 vs Ctr.

### The role of Dkk-3 mediated-apoptosis and the effects of TLR-4 pathway activation in the pathogenesis of GBM

**Figure 10:**
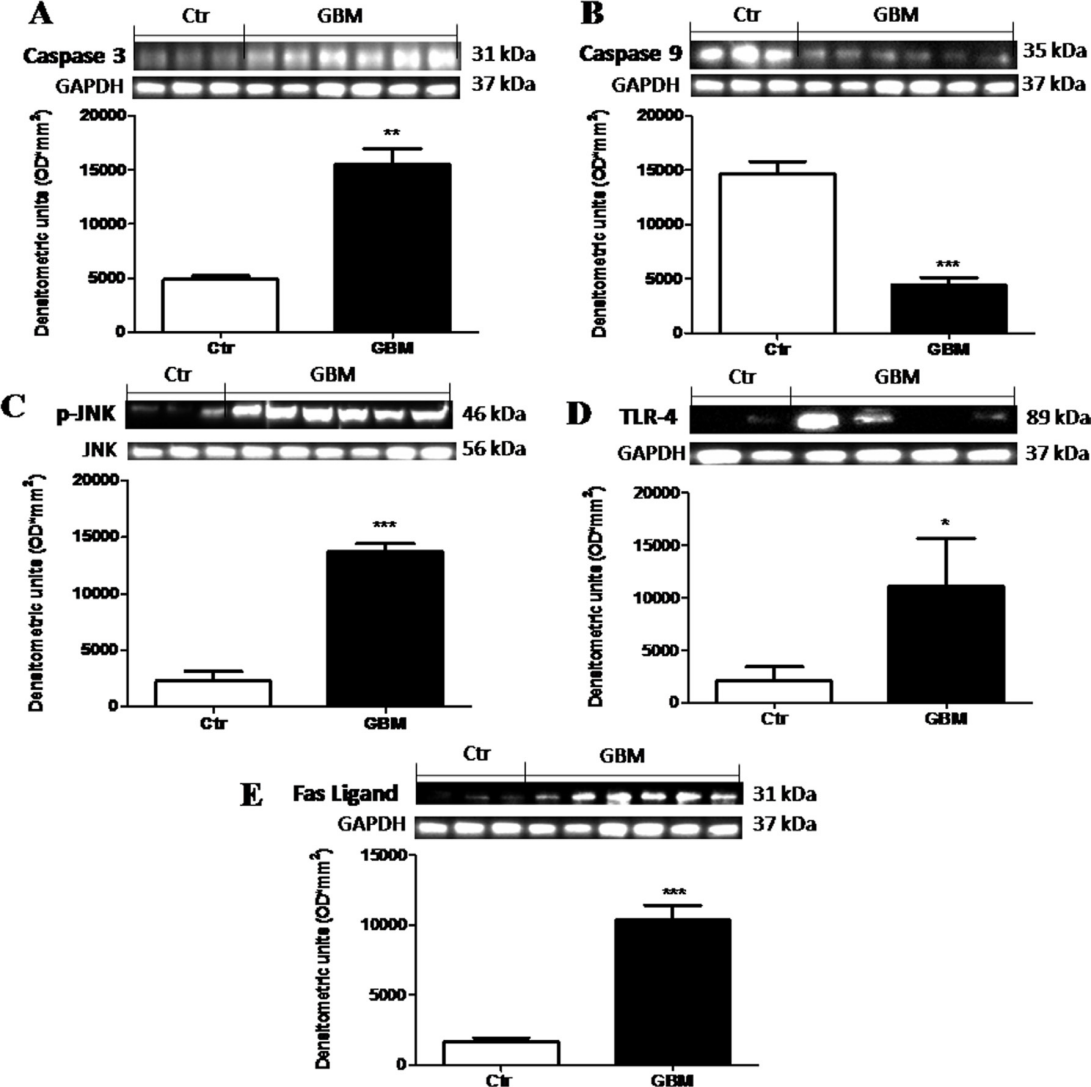
The role of Dkk-3 and TLR-4 pathway activation in the pathogenesis of GBM Differential expression of Caspase-3 was observed in the GBM patients with some of this expressing greater levels respect to basal expression in control tissue (**A**), while Caspase-9 levels were consistently lower in human tumor than in control tissue (**B**). Moreover, an increased expression levels of p-JNK was observed in tumor samples compared to healthy samples (**C**). The TLR-4 expression was significantly increased in GBM patients tissue (**D**) respect to control brain tissue (**D**). Also, FasL expression was notably higher in GBM samples (**E**). Each data are expressed as mean ± SEM of patients. *T* test, Unpaired *T* test. ^*^p < 0.05, ^**^p < 0.01 and ^***^p < 0.001 vs Ctr. Caspase 3, caspase 9 and fas-ligand were detected in the same stripped membrane (**A**, **B** and **E**) Therefore, the ubiquitary GAPDH band is the same for all three antibodies.

Alteration in the normal mechanism for programmed cell death plays an important role in the pathogenesis and progression of GBM patients [17]. So, to testify how the apoptotic processes could modulate GBM, we analyzed the apoptotic Caspase-3 and the anti-apoptotic Caspase-9 proteins by Western Blot analysis. We observed that caspase-9 was consistently lower in human tumor than in control tissue (Figure 10B). Differential significant expression of Caspase-3 was observed in the GBM patients respect to basal expression in control tissue (Figure 10A). On the contrary, GBM patients showed significant lower expression of Caspase-9 respect to control tissue (Figure 10B). Moreover, as many studies have demonstrated that JNKs are expressed and activated in the majority of GBM cases and posit that the JNK/c-Jun axis enhanced GBM cell sensitivity via stimulation of the apoptotic pathway [18], we evaluated p-JNK expression in GBM patients tissue. An increased expression levels of p-JNK was observed in tumor samples compared to healthy samples (Figure 10C). Furthermore, triggering TLRs to generate an immune response is therefore a primary goal in immunotherapy for cancer in general. Previous studies [19] proposed that TLR-4 expression, increased in human GBM but without however highlighting the mechanism of action. In this study, by Western Blot analysis, we confirmed that TLR-4 was significantly increased in GBM patients tissue (Figure 10D) respect to control brain tissue (Figure 10D), suggesting that the use of a TLR-4 antagonist could be able to induce anti-proliferative and antimigratory response in glioma tumors. Moreover, as FasL is implicated in GBM growth and invasion through the induction of apoptosis in infiltrating lymphocytes, which facilitate the evasion of the immune system by the tumor [17] we evaluated FasL expression by Western Blot analysis, observing a significant increase of FasL expression in human GBM samples respect to control samples (Figure 10E). Each data are expressed as mean ± SEM of patients. *T* test, Unpaired *T* test. ^*^p < 0.05 ^**^p < 0.01 and ^***^p < 0.001 vs Ctr.

## DISCUSSION

GBM is been designated by the World Health Organization as a grade IV cancer [20], characterized by rapid cell proliferation, treatment resistance and abundant angiogenesis [21]. The current treatments for GBM patients, can prolong survival for several months. Many newer agents, with varied treatment mechanisms, have failed in clinical studies. Clearly, better therapies that collectively target different aspects of glioma pathogenesis are needed [22]. The Wnt signaling pathway, a family of highly conserved secreted signaling molecules, has more recently become a central topic for study in cancer biology, specially pointing the potential of Wnt as a multi-faceted target in malignant glioma therapy [23].

A recent work demonstrated that Dkk-3 has distinct roles in regulating the Wnt pathway depending on the cell types examined, acting as a critical antagonist of the Wnt/beta-catenin signaling pathway [24]. Our results demonstrated that Dkk-3 was significantly reduced in glioma cell lines and in human GBM samples, observing lower levels in malignant human glioma cell lines (U-138MG and A-172), so underling the down regulation of Dkk-3 in growth. Loss of Dkk-3 expression is particularly observed in some types of cancer and, in *in vitro* studies was revealed that the impact of Dkk-3 on tumor growth is mediated by regulation of apoptosis [25]. It is known that highly invasive cancer cells are protected from apoptosis by increasing of various anti-apoptotic molecules including Caspase-9 protein [26]. Consistent with these findings, in our study we observed a higher expression of Caspase-9 rather than the apoptotic Caspase-3, in human GBM cell lines respect to control cell, suggesting low apoptotic activity in these tumor cells and underling that the antiproliferative property of Dkk-3 was due to the modulation of caspase-dependent apoptosis. High-expression levels of active Caspase-3 have been previously shown to be associated with an apoptotic phenotype of cells in GBM patient samples [27]. The examination of the data showed significantly greater expression of Caspase-3 in GBM patient samples compared with control tissue, while levels of Caspase-9 in all GBM resections were consistently lower than the standard control. Apoptosis is a basic biological process that promotes survival of the organism at the expense of individual cells and many studies observed an increased expression of FasL is involved in gliomagenesis [11]. In this work, we confirmed a significant expression of FasL in GBM patient’s samples.

Multiple complex pathways occur in the eukaryotic cells for the surveillance and repair of genetic material and cell cycle control. The intrinsic resistance to genotoxic therapies of malignant GBM cells could therefore be explained by their ability to stop growth and survive in a quiescent state and by the involvement of an enhanced DNA damage signaling [11]. Tail length observed in GBM human cell line compared to control cells was a considerable example of DNA damage, detected by Comet assay analysis.

Although the regulatory mechanisms underlying Wnt/Dkk-3 – induced apoptosis remain to be elucidated, Abarzua *et al.*, [28] showed that overexpression of Dkk-3 selectively induced apoptotic cell death in human prostate cancer cells via activation of c-Jun-NH2-kinase (JNK). In our study, we revealed that the JNK pathway is more consistently activated in human GBM cells than in control cell lines, and it is notably expressed in GBM tissues, confirming that the activation of JNK has a role in promoting the tumor growth.

Malignant gliomas present a therapeutic challenge because they are protected from cytotoxic chemotherapy by a heterogeneously permeable BBB. claudin-5, in various pathological tumor processes, regulated the change in endothelial or epithelial permeability, exercising a protective role of BBB to maintain its integrity [29]. In our work we observed, for the first time, a significantly reduction of claudin-5 expression levels in GBM human cell lines, further confirmed in GBM tissues.

GBM tumors activate and mobilize the innate immune system through activation of monitors including TLRs [30]. TLR-4 is the first identified member of the TLRs family expressed in many type of tumors and accumulating evidences demonstrated that the activation of TLR-4 in tumor microenvironment can not only boost the anti-tumor immunity but also give rise to immune surveillance and tumor progression [31]. In *in vitro* studies, on U-138, A-172, LN-18 and LN-229 GBM human cell lines, we observed a significant co-localization of TLR-4 and Dkk-3, demonstrating their effective role in GBM growth which could represent a possible protective way from tumor. Moreover, to better understand TLR-4 role on tumor growth, we demonstrated in *in vivo* study that the absence of TLR-4 strongly inhibited the growth of U-87 tumor xenografts, suggesting that the genetical absence of TLR-4 lead to a greater resistance to the tumor onset and development. The protective effect role of TLR-4 absence was also confirmed in our *clinical* study, with notably expression of TLR-4 in human GBM samples, pointing out a critical role of TLR-4 as biomarker of tumor metastasis and prognosis. Furthermore, TLRs activation has been demonstrated to downregulate the canonical Wnt signaling pathway, blocking at the early steps of the molecular cascade [11]. In this study, we demonstrated that TLR-4 gene deficiency preserved Dkk-3 and claudin-5 expressions and promoted apoptosis process, so determining a slowing of tumor growth. Moreover, on the basis which TLR-4 signaling pathway leads to the expression of Wnt and is the primary immune response, it may be prudent to conclude that a downregulation of TLR-4 pathway could limit GBM spreading modulatingWnt/Dkk-3/claudin-5 axis.

## MATERIALS AND METHODS

### *In vitro* studies

#### Cell lines

The normal human astrocytes cell line NHA (NHA-Astrocytes AGM LONZA^®^ CC-2565™ Homo sapiens astrocytes) and the human GBM cell lines: U138MG (U-138 MG ATCC^®^ HTB-16™ Homo sapiens brain glioblastoma), A172 (A-172 [A172] ATCC^®^ CRL-1620™ Homo sapiens brain glioblastoma), LN18 (LN-18 ATCC^®^ CRL-2610™) and L229 (LN-229 ATCC^®^ CRL-2611™) were purchased from American Type Culture Collection (ATCC) (Manassas, VA, USA). NHAcell line was cultured in 25 cm^2^ flask withCC-3186 AGM™ BulletKit™ (Kit which contains a 500 ml bottle of ABM™, (CC-3187) and AGM™ SingleQuots™ (CC- 4123) at 37° C in 5% CO_2_. The GMB human cell lineswere cultured in 75 cm^2^ flask withrespectively:ATCC-formulated Eagle’s Minimum Essential Medium (Catalog No. 30-2003) for U-138MG and ATCC-formulated Dulbecco’s Modified Eagle’s Medium (Catalog No. 30-2002) for A-172, LN-18 and LN-229 cell lines, supplemented with fetal bovine serum to a final concentration of 10% at 37° C in 5% CO_2_.

### Western blot analysis

For the preparation of total extracts from NHA, U138MG, A-172, LN-18 and LN-229 cell lines, cells were trypsinizedas previous described [13]. The filters were probed with specific Abs: anti-Dkk-3 (1:500, Abcam), anti-claudin-5 (1:500, Santa Cruz Biotechnology), anti p-JNK (1:500; Santa Cruz Biotechnology), anti-Caspase 3 (1:500; Millipore) and anti-Caspase 9 (1:500; Millipore) overnight at 4° C. The day after the membranes were incubated with a specific peroxidase-conjugated secondary antibody (Pierce, Cramlington, UK) for 1 h at room temperature and were analyzed by the enhanced chemiluminescence (KPL, USA). Protein signals were quantified by scanning densitometry using a bio-image analysis system (Bio-Profil, Milan, Italy) and the results were expressed as relative integrated intensity compared to controls. GAPDH (1:500, Santa Cruz Biotechnology) and JNK (1:500, Santa Cruz Biotechnology) were used to confirm equal protein loading and blotting. Signals were detected with enhanced chemiluminescence (ECL) detection system reagent according to the manufacturer’s instructions (Thermo, USA). The relative expression of the protein bands was quantified by densitometry with BIORAD ChemiDocTMXRS+software. Images of blot signals (8 bit/600 dpi resolution) were imported to analysis software (Image Quant TL, v2003).

Some antibodies were re-probed on the same blot by using stripping method where the membranes were incubated with a mixture of SDS, glycine, and detergents, two times for 20 min. This method let to detect multiple protein targets within a single membrane, since the 1°Ab was moved away from its target proteins, allowing other 1°Ab to be added [32].

### Immunofluorescence analysis

A-172 cells were processed for immunofluorescence analysis as previous described [33]. Cells were incubated with Dkk-3 and TLR-4 primary antibodies (diluted 1:100 in PBS with 1% BSA) for 2 h at RT, washed with PBS, and incubated with Alexa Fluor 594-conjugated anti-rabbit IgG or Alexa Fluor 488-conjugated anti-mouse IgG (Thermo Fisher, 1:1000 dilution in PBS with 1% BSA) for 1 h at RT. After several washes for 15 min (total) with PBS, cell nuclei were visualized with DAPI. Sections were observed using fluorescence microscope and photographed at 40× magnification using a Leica DM2000 microscope (Leica). All images were digitalized at a resolution of 8 bits into an array of 2560 × 1920 pixels. Digital images were cropped and figure montages prepared using Adobe Photoshop 7.0 (Adobe Systems; Palo Alto, CA).

### Alkaline (pH>13) comet assay

The alkaline microgel electrophoresis or “comet assay” was carried out to determine the extent of cellular DNA damage. A-172 cells were washed with ice-cold PBS. Following centrifugation cell pellets were resuspended in PBS (2 × 10^6^ cells/ml). Briefly, a 20 μl aliquot of cells (2 × 10^3^ cells/μL) was added to 180 μl of molten LM Agarose (0.7% low-melting agarose). After mixing, the sample was pipetted into an area of the comet slide. The slide was incubated at 4° C for 15 min to accelerate gelling of the agarose disc and then transferred to pre-chilled lysis solution (1.5 M NH4Cl, 10 mM EDTA, 100 mM NaHCO_3_) for 30 min at 4° C. Denaturation was performed in an alkali solution (0.3 M NaOH, 1 mM EDTA) at room temperature for 30 min, shielded from light. The slide was then transferred to TBE (300 mMNaOH, 1 mM EDTA) for 10 min to neutralize. Electrophoresis was conducted in a horizontal chamber using fresh TBE at 25 V (0.96 V/cm, approximately 300 mA) for 15 min. The slide was then fixed in ice-cold, 100% ethanol for 5 min, air-dried, and finally stained with 30 μl of 20 μg/ml ethidium bromide. Coded slides were viewed using a fluorescence microscope and the length of the whole comet (head and tail) was measured using image J software. In all cases percentage of DNA in the Comet tail (%TDNA) was used as a DNA damage parameter. Results were reported as Tail Length, which is indicative of the presence of DNA damage, expressed as mean of the 50 cells scored.

### In vivo

#### Cell line

Human glioblastoma cell line U-87 MG (U-87 MG ATCC^®^ HTB-14™ Homo sapiens brain Likely glioblastom) was obtained from ATCC (American Type Culture Collection, Rockville, MD, USA). U-87 MG cells were cultured in 75 cm^2^ flasks with ATCC-complete culture medium available as ATCC^®^ Catalog No. 4X.

### Animals

4–16-week-old male TLR4^−/−^ and C57BL/6J wild-type mice (WT) were purchased from Jackson Laboratory (Bar Harbor, Maine, United States) and maintained in microisolatorcages under pathogen-free conditions on a 12- hour light/12-hour dark schedule for a week. Standard laboratory diet and tap water were available ad libitum. The University of Messina Review Board for the care of animals approved the study. Animal care was in compliance with Italian regulations on protection of animals used for experimental and other scientific purposes (Ministerial Decree 16192) as well as with the Council Regulation (EEC) (Official Journal of the European Union L 358/1 12/18/1986).

### Experimental design

Tumor induction was performed as previous described [11]. After housing for a week, the mice (*n* = 12) were inoculated subcutaneously with 3•10^6^ glioblastoma U-87 MG cells in 0.2 ml PBS and 0.1 ml Matrigel (BD Bioscience, Bedford, MA). After injection, the mice were monitored for four weeks. After this period, the mice were sacrificed and their tumors were excised and processed for histology. Dimensions (length and width) of tumors were measured using a digital caliper, and the tumor burden was calculated using the following formula: 0.5 × length × width. Mean weight of mice at initiation of study and termination of study did not differ significantly between the groups. The tumor size was measured every four days for 28 days. The tumor volume was calculated using an empirical formula, V = 1/2 × [(the shortest diameter) 2 × (the longest diameter)].

### Histology

Mice were sacrificied at 28 days after infection and tumor were recised and processed for histological analysis. Tumor samples sections of 7-μm thickness were processed and were evaluated by an experienced histopathologist. Sections were then deparaffinized with xylene, and then stained with hematoxylin and eosin. All sections were studied using an Axiovision Zeiss microscope (Milan, Italy).

### Immunoistochemical analysis

For immunohistochemical localization of Dkk-3 and claudin-5, the sagittal sections of 7-μm thickness were processed as previous described [11] and incubated overnight with one of the following primary antibodies (all dilutions in PBS): monoclonal anti Dkk-3 (1:100, Abcam), and anti-claudin-5 (1:500, Santa Cruz Biotechnology). Photomicrographs were assessed by densitometric analysis using Imaging Densitometer (AxioVision, Zeiss, Milan, Italy).

### TUNEL assay

To determine the extent of intracerebral neuronal cell death, TUNEL was performed using a “Fluorescein *In Situ* Cell Death Detection Kit” (Roche Diagnostics GmbH, Mannheim, Germany), according to the manufacturer’s instructions. Briely tissue samples from the U-87 MG xenografted mice were fixed with 4% paraformaldehyde, cut into 7-μm thickness sections and processed as previous described [11]. After washing in PBS (three times for 3 min), sections were incubated in ice-cold ethanol-acetic acid solution (2:1) for 5 min at −20° C. Thereafter, they were washed in PBS and incubated in a permeabilization solution with 3% Triton X-100 in PBS for 60 min at RT, then incubated with the TdT enzyme in a reaction buffer containing fluorescein-dUTP for 90 min at 37° C. Negative control was performed using only the reaction buffer without TdT enzyme. Positive controls were performed by digesting equal brain sections with DNase grade I solution (500 U/ml; Roche) for 20 min at RT and always kept separate from the other samples thereafter. After labelling, the sections were washed again in PBS and to visualize the unstained (TUNEL-negative) cells, the sections were covered with Vectashield^®^ mounting medium for fluorescence with DAPI (Vector). All samples were evaluated immediately after staining using an Axioskop 40 fluorescence microscope (Zeiss, Germany) at 460 nm for DAPI and 520 nm for TUNEL fluorescence and analyzed by Alpha digi doc 1201 software (Alpha Innotech, San Leandro, CA, USA).

### Clinical studies

#### Patients and samples

Our study includes 30 cases of glioblastoma of IV histological grading, including 9 women and 21 men, aged between 40 and 65 years with an average age of 52.5%. All patients were operated on at the Unit of Neurosurgery, Department of Biomedical and Dental Sciences and Morphofunctional Imaging, University of Messina, Messina, Italy. All patients had provided informed consent and tumour collection was approved by the Regional Ethical Board at the University of Messina. All biopsies were primary GBM based on neuropathological diagnosis and biomolecular analysis. Surgical specimens were obtained from 30 brain tumors of patients who had undergone surgery between January 2017 and July 2017. All the brain tumor tissues were collected during craniotomies for their resection or during biopsy; the tissues were immediately stored in a deep freezer at –70° C prior to experimental study. Histopathologic evaluation of the brain tumor tissues was performed by a neuropathologist and this was based on the World Health Organization Grade 4 classification of gliomas [34]. By reviewing patient records and contacting patients, families and referring physicians, the following information was gathered: age, sex, tumor location, clinical presentation, and surgical data.

### Experimental groups

Glioblastoma and negative control samples were divided into 2 groups:

1. Control group (Ctr): healty brain tissues were processed and used as negative control
2. GBM samples: glioblastoma tissues from patients were processed and used as positive control

### Western blot analysis

Total cellular and tissue proteins of brain tissue were processed as previously described [13]. The filters were probed with specific Abs: anti-Dkk-3 (1:500; Millipore) or anti-claudin-5 (1:500; Abcam) or anti p-JNK (1:500; Santa Cruz Biotechnology) or anti-Caspase 3 (1:500; Millipore) or anti-Caspase 9 (1:500; Millipore) or anti-TLR-4 (1:500; Santa Cruz Biotechnology) or anti-Fas Ligand (FasL) (1:500, Abcam) in 1 × PBS, 5% w/v non fat dried milk, 0.1% Tween-20 at 4° C, overnight. The day after the membranes were incubated with a specific peroxidase-conjugated secondary antibody (Pierce, Cramlington, UK) for 1 h at room temperature and were analyzed by the enhanced chemiluminescence (KPL, USA). Protein signals were quantified by scanning densitometry using a bio-image analysis system (Bio-Profil, Milan, Italy) and the results were expressed as relative integrated intensity compared to controls. GAPDH (1:500, Santa Cruz Biotechnology) and JNK (1:500, Santa Cruz Biotechnology) were used to confirm equal protein loading and blotting. Signals were detected with enhanced chemiluminescence (ECL) detection system reagent according to the manufacturer’s instructions (Thermo, USA). The relative expression of the protein bands was quantified by densitometry with BIORAD ChemiDocTMXRS+software. Images of blot signals (8 bit/600 dpi resolution) were imported to analysis software (Image Quant TL, v2003).

Some antibodies were re-probed on the same blot by using stripping method where the membranes were incubated with a mixture of SDS, glycine, and detergents, two times for 20 min. This method let to detect multiple protein targets within a single membrane, since the 1°Ab was moved away from its target proteins, allowing other 1°Ab to be added [32].

### Statistical analysis

All values, in the figures, were evaluated as mean ± SEM. The results were examined by ANOVA One-way analysis of variance followed by a Bonferroni post-hoc test for multiple comparisons and *T* test (Unpaired test); *p* values < 0.05 were considered statistically significant.

## CONCLUSIONS

The data obtained in this study demonstrated that the regulation of Dkk-3 confirmed the caspase-mediated apoptosis involvement in tumorigenesis process while the modulation of claudin-5 highlited the role of BBB to maintain its integrity in tumor environment. Furthermore, we demonstrated, for the first time, an interesting TLR-4/Wnt modulation, suggesting that combined therapies that simultaneously enhance Wnt/Dkk-3/claudin-5 activity and inhibit TLR-4 pathway, might shift the balance from tumor growth stasis to cytotoxic therapeutic responses. Taken together, a more clarification between the Wnt/Dkk-3/claudin-5 signaling and the TLRs pathway will be better investigated to find new possible molecular targets for future therapeutics protocols.

## Abbreviations

GBMs: Glioblastomas
Wnt: Wingless signaling
Dkk-3: Dickkopf protein-related protein 3
TJ: Tight Junctions
TLRs: Toll-like receptors
TLR-4 KO: Toll-like receptor 4 knock out
PAMPs: Pathogen Associated Molecular Patterns
NHA: Normal Human Astrocytes cell line
JNK: c-Jun-NH2-Kinase
p-JNK: phospho-c-Jun-NH2-Kinase
GAPDH: Glyceraldehyde 3-phosphate dehydrogenase
ECL: EnanchedChemioluminescence
MR: Magnetic Resonance
BBB: Brain Barrier Blood
